# Application of the Self-Assembling Peptide Hydrogel RADA16 for Hemostasis during Tonsillectomy: A Feasibility Study

**DOI:** 10.3390/jfb15090271

**Published:** 2024-09-18

**Authors:** Joshua Michaels, Anna I. Kaleva, Laura Bateman, Oliver Wakelam, Joanna Stephens

**Affiliations:** 1 Department of Otolaryngology, North West Anglia NHS Foundation Trust, Peterborough PE3 9GZ, UK; 2 Department of Otolaryngology, Cambridge University Hospitals NHS Foundation Trust, Cambridge CB2 0QQ, UK; anna.kaleva@nhs.net; 3 Royal Hospitals Bath NHS Foundation Trust, Bath BA1 3NG, UK; laura.bateman2@nhs.net; 4 Norfolk and Norwich University Hospitals NHS Foundation Trust, Norfolk NR4 7UY, UK; oliverwakelam@nhs.net; 5 East and North Hertfordshire NHS Trust, Stevenage SG1 4AB, UK; joannastephens@nhs.net

**Keywords:** tonsillectomy, RADA16, hydrogel, hemostasis, readmission, re-operation, bleeding, wound healing

## Abstract

Tonsillectomy is a common surgical procedure but carries a high risk of readmission for secondary bleeding and pain. This study evaluated the feasibility and effectiveness of using the hemostatic self-assembling peptide hydrogel RADA16 (PuraBond, 3-D Matrix SAS; Caluire et Cuire, France) to control bleeding from the tonsillectomy wound bed. Readmission/re-operation rates were compared between a prospective case series of 21 primarily adult tonsillectomy patients treated with topical RADA16 and an untreated historical Control group of 164 patients who underwent tonsillectomy by 10 surgeons at a single tertiary hospital in the UK between March 2019 and June 2022. Cumulative readmission rates for any reason were 2-fold elevated in Control subjects (18.9%; *n* = 31/164 subjects) compared to patients treated intra-operatively with RADA16 hemostatic hydrogel (9.5%; *n* = 2/21) (*p* = 0.378). Readmission rates for postoperative bleeding were 3-fold higher in Controls (14.6%; *n* = 24/164 subjects) than in the RADA16-treated group (4.8%; *n* = 1/21) (*p* = 0.317). A similar rate of retreatment for pain was recorded in the Control (4.3%; *n* = 7/164) and RADA16 (4.8%; *n* = 1/21) groups (*p* = 0.999). Two Control subjects (1.2%) required re-operation for recalcitrant bleeding; no RADA16 subject (0.0%) required re-operation for any reason. No device-related adverse events occurred in the RADA16 group. Surgeons were pleased with the easy learning curve and technical feasibility associated with intra-operatively administering RADA16 hemostatic hydrogel. Intra-operative hemostasis using RADA16 peptide hydrogel was straightforward and was associated with a trend of 3-fold lower rates of readmission for postoperative bleeding events than untreated Control subjects.

## 1. Introduction

Tonsillectomy is the surgical excision of the palatine tonsils, lymphoid tissue along the lateral wall of the oropharynx that is covered in respiratory epithelium and invaginated to create crypts. Tonsillectomy is one of the most commonly performed surgical procedures in the UK, accounting for an estimated 17% of the otorhinolaryngology elective workload [1]. Because of the highly vascular nature of the tonsils, surgical removal carries risk of post-tonsillectomy hemorrhage (PTH) [2]. A 2023 randomized trial performed in 27 UK hospitals reported an adult post-tonsillectomy readmission rate of 19% for bleeding [3]. Thus, PTH is the most common cause for tonsillectomy patients returning for subsequent medical care.

RADA16 is an ionic-complementary self-assembling peptide whose chemistry and clinical utility were recently reviewed in detail [4]. Briefly, the 16-residue synthetic peptide contains repeated 4-amino acid sequences containing alternating positively and negatively charged amino acids (i.e., “R” positively charged arginine; “A”, hydrophobic alanine; and “D”, negatively charged aspartic acid residues). RADA16 monomers have a β-strand periodicity and take advantage of their repeated charge patterns and hydrophobic interactions and hydrogen bonding to spontaneously reorganize into long nanofibers with an interlinked β-sheet structure [4,5,6]. Upon exposure to the physiological pH and ionic environment of body fluids such as blood and interstitial fluid, the solubilized nanofibers interlink to form mesh-like hemostatic hydrogels. The CE-marked RADA16 products (PuraBond, PuraShield, and PuraStat) are identical topical hemostatic agents that form a barrier to stop bleeding and maintain a moist wound environment to support optimal healing [7,8,9]. The formulation is a sterile viscous 2.5% (*w*/*v*) aqueous solution of the repeating peptide RADA16 that is delivered by syringe. Upon exposure to bodily fluids, the RADA16 nanofibers spontaneously and reversibly self-assemble into a gel-like biocompatible hydrogel wound cover that naturally degrades to constituent amino acids that are eventually resorbed as wound healing progresses. These identical product formulations received FDA clearance in the US as hemostatic wound-healing devices with dermal (PuraDerm), gastrointestinal (PuraStat), and sinus applications including the prevention of adhesion formation and as an adjunct to wound healing (PuraSinus, now branded as PuraGel) [7,8,9]. It is also licensed for similar use in Australia.

Because multiple contemporary clinical studies demonstrated the utility of using RADA16 as an intraoperative hemostatic wound dressing for treating gastrointestinal mucosal lesions in the esophagus [10], stomach [11], colon [12], and nasosinus cavity [12,13], and the wound bed after transoral robotic excision of oropharyngeal squamous cell cancers [14], we hypothesized that RADA16 may be an appropriate treatment to prevent bleeding at the post-tonsillectomy pharyngeal mucosal wound bed.

This study evaluated the feasibility of using RADA16 as an intra-operatively administered topical hemostatic agent during tonsil resection in adults and assessed the relationship between RADA16 use and readmission rates for surgical site PTH.

## 2. Materials and Methods

### 2.1. RADA16 Hydrogel Formulation

The PuraBond^®^ device (3-D Matrix SAS, Caluire et Cuire, France) is a viscous acidic aqueous solution containing 2.5% RADA16, a synthetic 16-amino acid peptide constructed with four repeating amino acids (arginine-alanine-arginine-aspartic acid)_4_. The same RADA16 formulations are CE-marked as Class III medical devices approved as topical hemostatic agent to control bleeding from small blood vessels and solid organ parenchyma, at suture lines along vascular anastomoses, and from vessels of the gastrointestinal mucosa after tissue repair or resection. A related product (also 2.5% aqueous RADA16), received FDA clearance in 2019 as an intraoperative hemostatic wound dressing that also functions to prevent adhesion formation and acts as an adjunct to wound healing after nasal surgery or trauma repair [9].

When applied intra-operatively, the RADA16 solution spreads across the wound surface and polymerizes into a protective transparent hydrogel. Aqueous RADA16 exhibits thixotropic (shear-thinning) properties that facilitate syringe/catheter delivery of a viscous solution that conforms to the wound bed topography before undergoing polymerization. This allows easy application in tight surgical fields. Upon contact with the physiological pH of body fluids, the peptide nanofibers of the acidic RADA16 solution physically crosslink to form a protective, hemostatic hydrogel wound covering. Its transparent nature permits continued visualization of the surgical site after application, which allows for monitoring the wound bed and/or suture lines for recalcitrant bleeding that might not be easily identifiable after using opaque topical hemostatic agents based on, for example, collagen, cellulose, or fibrin [15]. The RADA16 formulation is non-toxic, biocompatible and non-irritating, and is enzymatically degraded and resorbed over time [4]. Because it is a synthetic peptide, there is no risk of microbial carryover or contamination that may be a consideration with organic or animal-derived hemostatic agents such as collagen and fibrin. The hydrogel does not swell during or after polymerization, as occurs with certain topical hemostatic agents (e.g., gelatin) [15], ensuring that delicate nearby structures such as nerves and microvasculature are not at risk of potential compression injury within often-confined surgical fields.

### 2.2. Study Overview

We report the clinical experience, surgical outcomes, and postoperative results of a consecutive case series of adult patients who underwent tonsillectomy, with or without RADA16 adjunctive hemostasis, performed by 10 surgeons at Lister Hospital (Hertfordshire, UK) between March 2019 and June 2022. As a service evaluation of an intervention being used within its license, no ethical approval was required from the UK Health Research Authority [16]. Patients were consented to tonsillectomy with PuraBond delivery at surgeon discretion according to current clinical practice, with no additional risk above the standard risks for tonsillectomy. This feasibility study was conducted under the auspices of the Associate Director of Research at East and North Hertfordshire NHS Trust (Hertfordshire, UK). Patients provided written informed consent to receive tonsillectomy with or without RADA16 application. All patient data were anonymized and treated with confidentiality according to the tenets of the Declaration of Helsinki.

### 2.3. Procedural Details

Patients underwent a standard pre-operative workup, and underwent general anesthesia induction, intubation, neck extension supported by a rolled towel under the shoulders, and Boyle–Davis mouth gag insertion with Draffin rod stabilization to visualize the oropharynx and stabilize the tracheal tube. After ascertaining endotracheal tube placement in the airway midline and verification that the airway was not obstructed or displaced, tonsillectomy was performed according to surgeon technical preference. Briefly, on each side, the initial incision was made at the superior pole with hemostasis of feeder vessels, followed by extracapsular cold dissection with bipolar hemostasis until the inferior pole was isolated, clamped, and the tonsil excised. Silk ties were placed on the inferior tonsillar poles and hemostasis was achieved with bipolar cauterization (diathermy) followed, in some patients, by application of RADA16 gel to the surgical wound. Tonsillectomy dissection was then repeated on the contralateral side.

For patients receiving RADA16, a single 5-mL tube was shared between both wound sites. After cleaning the wound bed with sterile gauze, RADA16 was applied as a thin, wide layer overlapping the approximate 2 × 2 cm wound bed for each tonsil and wherever bleeding was noted. Typically, 0.5–1.0 mL of hydrogel was used per tonsil bed, with repeat application to bleeding sites permitted at surgeon’s discretion. After tonsillectomy with hemostasis was achieved, the postnasal space was suction aspirated, the oral cavity thoroughly examined for anomaly or injury, and the patient was monitored in recovery for 4–6 h with surgeon review during the first hour. Patients were discharged the same day and advised to return to hospital if spitting up more than 2 teaspoons of blood. Postoperatively, benzydamine hydrochloride (Difflam; Viatris Inc., Canonsburg, PA, USA) rinse and oral acetaminophen (paracetamol), and ibuprofen were prescribed for 2 weeks, with oral codeine analgesia prescribed for the first 3 days, if needed. No follow-ups were routinely scheduled.

### 2.4. Outcome Measures

The primary study goal was to assess surgeon impressions with the feasibility of incorporating RADA16 hemostasis into the tonsillectomy protocol, and to collect their impressions on its utility as a transparent intraoperative topical hemostatic agent. Feasibility was evaluated using a surgeon satisfaction survey. Main effectiveness outcomes were the readmission and reoperation rates for any reason, for recalcitrant PTH, and for pain management. The primary safety outcome was the incidence of procedure- or device-related adverse events (AEs) observed by the surgeon or reported by the patient.

### 2.5. Statistical Analysis

Continuous data are shown as mean ± SD, with differences evaluated by paired two-tailed *t*-tests. Categorical data are presented as *N* (%) and were compared using two-tailed Fisher exact test. Statistical significance was defined as *p* < 0.05. Prism v.5.0 statistics and graphing software (GraphPad Software, La Jolla, CA, USA) was used.

## 3. Results

During the study period, a total of 21 and 164 patients underwent tonsillectomy, with and without RADA16 application, respectively, at our medical center. Patient demographics and indication for surgery are provided in Table 1. All RADA16 subjects had day-case surgery, whereas 7.9% of Controls were overnight admissions. Recurrent clinical tonsillitis was the predominant indication for surgery in both groups, with histological, quinsy, and sleep apnea indications being less common.

**Figure 1:**
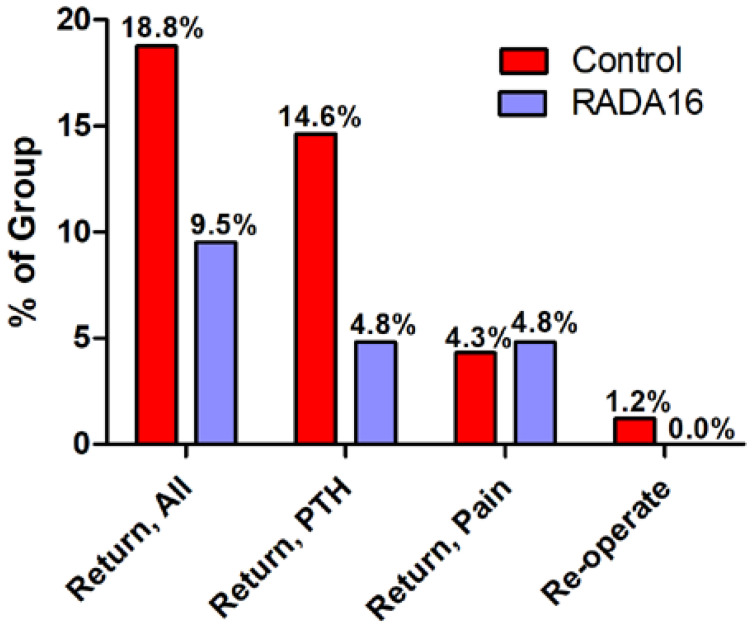
Readmission and re-operation rates after tonsillectomy with and without RADA16 hemostatic hydrogel application to the tonsil wound beds. The incidence of returns to hospital for any reason were 2-fold higher in untreated Controls than in the RADA16 group, with the rate of return for post-tonsillectomy hemorrhage (PTH) being 3-fold higher in Controls. Readmission rates for ongoing pain were similar in both groups. None (0.0%; *n* = 0/21) of the RADA16-treated individuals required a return to the operating theater, whereas 1.2% (*n* = 2/164) of Control subjects needed re-operation to control bleeding.

A 2-fold larger fraction of the Control group (18.9%, *n* = 31/164 subjects) than RADA16 recipients (9.5%, *n* = 2/21 subjects) returned to hospital for any reason after discharge, although this trend did not attain statistical significance (*p* = 0.378) (Figure 1). Post-tonsillectomy hemorrhage was the predominant reason for seeking postoperative care in the Control group (77.4%, *n* = 24/31, of readmissions); PTH accounted for 50% (*n* = 1/2) of all RADA16 group readmissions. The readmission rate for PTH trended 3-fold higher in Controls (14.6%, *n* = 24/164 subjects) compared to subjects who received RADA16 hemostasis (4.8%, *n* = 1/21; *p* = 0.317) (Figure 1). Of the 24 Control subjects who returned because of bleeding, 8.3% (*n* = 2/24) required a return to the operating theater; the single RADA16 subject who was readmitted for ongoing bleeding (postoperative Day 5) did not require re-operation and was satisfactorily managed with continued anti-inflammatory and analgesic therapy with a new prescription for antibiotics and 3% hydrogen peroxide gargling.

Pain was the second most-common reason for readmission in the Control group, accounting for the remaining 22.6% (7/31) of total readmissions. Pain was the causative factor for readmission of a single RADA16 subject (1/2 returnees). No subject in the RADA16 group experienced an AE that was known or suspected to be device-related.

Four surgeons were queried about feasibility parameters and satisfaction associated with intra-operative RADA16 use. Overall satisfaction was rated as 4.25 of 5.0 possible points, with high scores for ease-of-use, hemostatic effectiveness, and the benefits of the gel’s transparent nature (Table 2).

## 4. Discussion

In this study, treating the post-tonsillectomy wound bed with hemostatic self-assembling RADA16 peptide hydrogel was associated with a trend towards reduced hospital readmissions for any reason (2-fold difference versus untreated Controls) and specifically with reduced admissions for recalcitrant bleeding events (3-fold difference). The tonsillar branch of the facial artery is the primary arterial blood supply, with the majority of venous drainage into the pharyngeal plexus [2,17]. The external palatine vein enters the tonsillar bed from the soft palate and this large vein is frequently the source of venous bleeding after tonsil excision. Postoperative bleeding is the most common cause of morbidity following tonsillectomy, due in part to this extensive vascularization by tributaries of the external carotid arterial and palatine vein drainage systems [17].

In the UK, an estimated 19% of persons ≥16-years-old who undergo tonsillectomy are readmitted for postoperative bleeding [1]; rates higher than previously reported in the literature [3,18]. Thus, finding ways to reduce the incidence of PTH is essential for improving patient outcomes and reducing readmission rates. Application of topical RADA16 to the tonsillectomy wound bed was demonstrated to be easily incorporated into the surgical protocols used by different surgeons, and may be a simple and minimally-invasive approach for reducing the incidence of postoperative rebleeding events.

Severe cases of PTH may require a return to the operating theater to achieve reliable hemostasis. No participant in the RADA16 study arm needed re-operation for any reason, including bleeding. The Control group’s re-operation rate in our study was 1.2%, all for bleeding, which is similar to rates observed in other large population studies of UK adults (e.g., 1.2–2.3%) [1,18,19]. It will be essential to confirm whether RADA16 treatment reduces the incidence of post-tonsillectomy re-operation for PTH in a larger prospective trial.

Because the bare tonsillectomy bed heals by secondary intention and is extensively innervated, pain can persist for up to 2–3 weeks after surgery, although it is typically maximal the first few days and wanes thereafter [20]. A similar 4–5% of patients in both study groups were readmitted for ongoing post-tonsillectomy pain. While RADA16 was shown to facilitate wound healing by supporting proliferation and differentiation of diverse cell types [4], including mucosal re-epithelialization [21,22], benefits to recovery in the current study were not reflected in reduced visits for postoperative pain. Self-assembling RADA16 hydrogels demonstrated utility as a drug reservoir and delivery system for cytokines and growth factors in experimental models of wound repair [4,5,6]. The RADA16 matrix’s functional utility may plausibly be expanded in the future for use as a medication depot for the sequestration and regulated release of topical anti-inflammatory and anesthetic agents to address post-tonsillectomy pain.

Hemostasis using RADA16 has multiple comparative benefits over other topical agents examined for treating the post-tonsillectomy wound bed [23]. In addition to staunching mild-to-moderate oozing blood flow, the biocompatible peptide hydrogel provides a moist wound environment and a protective interlinked fibrillar scaffold that resembles the native extracellular matrix [24]. The hydrogel is cleared by endogenous enzymatic action to non-toxic amino-acid metabolites over days to weeks, depending on the physical milieu of the application site [4]. Because RADA16 is a synthetic product, there are no concerns associated with possible biocontaminants or immunogenicity that may exist with human- or animal-based products [25,26]. Syringe and catheter delivery is straightforward and can be repeated at operator discretion. The shear-thinning/thixotropic properties permit delivery of RADA16 solutions through small-diameter applicators and allow the product to easily spread across and cover the wound topography before undergoing gelation. Because RADA16 nanofiber diameter is smaller than the wavelength of visual light, both the applied solution and resulting polymerized hydrogel wound covering are transparent, thereby permitting continuous visual assessment of the wound bed that is unavailable with opaque topical wound treatments [4,27]. Although the current study protocol did not include photographing RADA16 application to the tonsil bed, prior publications include images in which gastrointestinal and nasosinopharangeal mucosal wound beds are clearly visible under the transparent topical hydrogel [4,10,11,12,13,14]. Surgeons appreciated the straightforward delivery process, quickly achieved hemostasis, and transparent nature of the RADA16 material that allowed clear visualization of continued or new bleeding.

Recent clinical studies explored using RADA16 as an intraoperative hemostatic wound dressing for lesions in the esophageal [10], gastric [11], colon [12], and nasosinus [12,13] mucosae. The RADA16 hydrogel also began exploration for treating the mucosal bed after transoral robotic excision of oropharyngeal squamous cell cancers [14]. This study extends these investigations by additionally demonstrating RADA16 peptide’s utility for treating the post-tonsillectomy oropharyngeal mucosal wound bed.

Although the trend of RADA16 treatment reducing the incidence of post-tonsillectomy bleeding events is encouraging, we acknowledge that this retrospective feasibility study was not sufficiently powered to characterize this effect. Additionally, results from our adult population are not directly generalizable to the pediatric population in which many tonsillectomies occur. Future prospective trials enrolling a larger patient population including pediatric participants will confirm and definitively quantify the relationship between RADA16 administration and the rate of rebleeding events following tonsil excision. Readmission rates for PTH vary widely across studies, and even across institutions within those studies [1,17,18]. Thus, it will be important to standardize clinical research protocols to identify factors such as surgical technique and patient characteristics that may be responsible for variations in post-tonsillectomy morbidity outcomes.

## 5. Conclusions

Intra-operative RADA16 application to tonsillectomy wounds is technically feasible and is associated with good hemostasis that can be continuously monitored through the transparent protective hydrogel covering it forms over the tonsil beds. The trends of reduced readmissions for PTH in RADA16-treated individuals to one-third the rate of untreated individuals and half the total readmission rate for any reason are promising and deserve confirmation in larger prospective studies.

## Figures and Tables

**Table 1:** Patient Information.

Parameter	Controls (*N* = 164)	RADA16 (*N* = 21)
Age, years, mean	32.0	23.3
Female, *n* (%)	111 (67.7%)	18 (85.7%)
Day surgery, *n* (%)	151 (92.1%)	21 (100%)
Indication, *n* (%)		
Tonsillitis	101 (61.6%)	17 (81.0%)
Histology	57 (34.8%)	1 (4.8%)
Quinsy	4 (2.4%)	3 (14.3%)
Obstructive sleep apnea	2 (1.2%)	0 (0.0%)

**Table 2:** Feasibility and surgeon satisfaction with intra-operative RADA16 hemostasis.

Parameter	Score, Mean Points *
Handling/delivery	4.75 ± 0.50
Hemostatic effectiveness	4.25 ± 1.00
Transparency benefits	4.25 ± 0.50
Ready-to-use format	4.50 ± 0.60
Overall satisfaction	4.25 ± 0.50

* Satisfaction was graded on a 1–5 point scale, with 1 = strongly dissatisfied, 3 = neutral, and 5 = strongly satisfied.

## Data Availability

The original contributions presented in the study are included in the article, further inquiries can be directed to the corresponding author.
